# Rapid Response Subunit Vaccine Design in the Absence of Structural Information

**DOI:** 10.3389/fimmu.2020.592370

**Published:** 2020-11-04

**Authors:** Danushka K. Wijesundara, Michael S. Avumegah, Julia Lackenby, Naphak Modhiran, Ariel Isaacs, Paul R. Young, Daniel Watterson, Keith J. Chappell

**Affiliations:** ^1^ School of Chemistry and Molecular Biosciences, The University of Queensland, St Lucia, QLD, Australia; ^2^ The Australian Institute for Biotechnology and Nanotechnology, The University of Queensland, St Lucia, QLD, Australia; ^3^ Australian Infectious Disease Research Centre, The University of Queensland, St Lucia, QLD, Australia

**Keywords:** Disease X, subunit vaccine, molecular clamp, pre-fusion conformation, membrane fusion protein, *Paramyxoviridae*, *Arenaviridae*, Coalition for Epidemic Preparedness Innovations

## Abstract

Prior to 2020, the threat of a novel viral pandemic was omnipresent but largely ignored. Just 12 months prior to the Coronavirus disease 2019 (COVID-19) pandemic our team received funding from the Coalition for Epidemic Preparedness Innovations (CEPI) to establish and validate a rapid response pipeline for subunit vaccine development based on our proprietary Molecular Clamp platform. Throughout the course of 2019 we conducted two mock tests of our system for rapid antigen production against two potential, emerging viral pathogens, Achimota paramyxovirus and Wenzhou mammarenavirus. For each virus we expressed a small panel of recombinant variants of the membrane fusion protein and screened for expression level, product homogeneity, and the presence of the expected trimeric pre-fusion conformation. Lessons learned from this exercise paved the way for our response to COVID-19, for which our candidate antigen is currently in phase I clinical trial.

## Introduction

Viral fusion proteins, which catalyse fusion between viral and cellular membranes during viral entry, are embedded in the virion membrane and displayed on the virion surface. Hence, they are primary targets of neutralizing antibody responses. Importantly, all viral fusion proteins on the virion surface, are found on the virion surface in a metastable “pre-fusion” form that is primed and ready to undergo a major conformational change following triggering by receptor engagement and/or by the low pH environment of endosomes (1). Refolding into this highly stable “post-fusion” form is what drives the process of virus-host cell membrane fusion. Similarly, purified or isolated viral fusion proteins are naturally unstable and trigger into the post-fusion conformation. The conformational changes involved in this transition can dramatically alter the surface topography of the fusion protein itself and hence their display of antigenic epitopes. Therefore, in order for vaccination to elicit an immune response that can prevent infection, the antibodies induced must recognize viral proteins in the active, pre-fusion conformation present on the virion surface to ensure efficient virus neutralization. Recent studies of human antibody responses to naturally acquired infections have clearly shown that the majority of neutralizing antibodies induced are those that recognize the pre-fusion form of these viral fusion proteins (2, 3).

**Figure 1:**
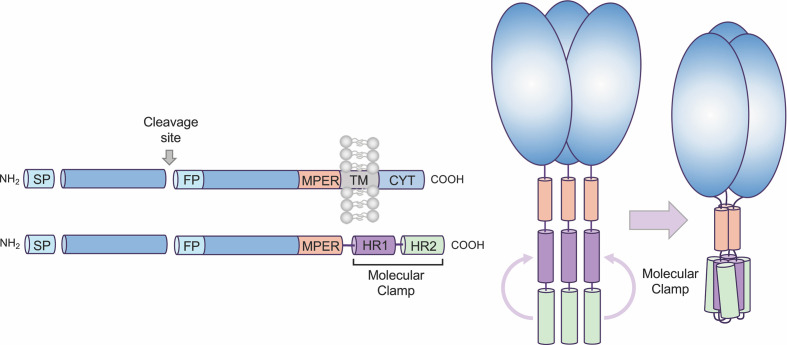
Overview of Molecular Clamp stabilized antigen design. Class I and III viral fusion proteins share common molecular features including a C-terminal transmembrane domain. To generate a soluble, secreted antigen the Molecular Clamp, comprising of hepad repeat (HR) regions 1 and 2 of human immunodeficiency virus (HIV)-1 glycoprotein 41 (gp41), is incorporated into recombinant viral glycoproteins in place of the C-terminal transmembrane/cytoplasmic region. We have also shown that selected modifications within the signal peptide (SP, replacement with a more efficient SP such as IgK SP), cleavage site/s (ablated or enhanced), fusion peptide (deletion), and deletion of the transmembrane domain adjacent to the membrane-proximal external region (MPER) to introduce the clamp domain can increase expression levels of soluble recombinant antigens.

We have developed a proprietary recombinant protein expression approach, Molecular Clamp, comprising a highly stable trimerization domain, that when incorporated into viral fusion proteins effectively constrain them in their native pre-fusion conformation (**Figure 1**). While generic trimerization motifs, such as GCN4 (an isoleucine zipper) and foldon (from the T4-phage fibritin), have previously been used for the stabilization of viral fusion proteins, additional modifying mutations and/or the inclusion of disulphide bridges have been required to achieve sufficient stability; as has been shown for Respiratory Syncytial virus (RSV) Fusion protein (F) (4), Human Immunodeficiency virus (HIV) glycoprotein 140 (gp140) (5), and Middle Eastern Respiratory Syndrome Coronavirus (MERS-CoV) Spike protein (6). Although these stabilized antigens show promise as vaccine candidates and are advancing into the clinic, such structure-based changes are not always compatible with rapid vaccine development against emerging viruses, as they require detailed knowledge of protein structure and significant time to identify and validate stabilizing mutations. In comparison, and as will be highlighted in the current study, the Molecular Clamp alone is able to facilitate pre-fusion stabilization without the need for labor-intesive, additional modifications, at least for class I virus fusion proteins. The Molecular Clamp is a trimerization motif of 80aa in length (~9.2 kDa) derived from N- and C-terminal heptad repeat (HR) regions of HIV-1 gp41 which self-assemble into a stable six-helical bundle structure that is critical for driving membrane fusion and cell entry of HIV-1 (7) (**Figure 1**). Furthermore, the enhanced stability of the Molecular Clamp facilitates rapid *in silico* antigen design based solely on a shared trimeric architecture which could ensure that immune responses to conformational epitopes that are unique to the pre-fusion form and essential for protection are elicited following vaccination of the subunit vaccine candidates developed using the Molecular Clamp.

All class I viral fusion proteins are trimeric and share a number of conserved features, including an N-terminal signal peptide, a C-terminal transmembrane domain, and a small cytoplasmic domain (**Figure 1**). Due to this shared architecture, the Molecular Clamp can be easily incorporated into recombinant viral glycoproteins in place of the C-terminal transmembrane/cytoplasmic region. While inclusion of the C-terminal Molecular Clamp trimerization domain is sufficient to achieve pre-fusion stabilization of a soluble trimeric protein, we have also observed that protein yield and homogeneity can be affected by antigen design. However our experiences with expression of recombinant viral fusion proteins from a number of different virus families have allowed us to identify a limited set of protein structural regions that can impact on yield and homogeneity, including **(i)** the N-terminal signal peptide, **(ii)** proteolytic cleavage site(s), **(iii)** the fusion peptide, and **(iv)** the membrane-proximal external region (MPER). Considering the importance of time within an emergency scenario, sequential optimization of these factors would not be possible. Instead, to ensure maximal yield downstream, we generate a panel of 20–30 antigens containing deletions and substitutions at sites based on modifications to the structural regions described above. This panel of antigens is then screened for protein expression, homogeneity and pre-fusion stabilization and an optimal design (or possibly small subset of designs) can be quickly selected for further preclinical development including immunogenicity analysis in pre-clinical models and simultaneous manufacturing process optimization.

In the current study, we present findings of two proof of concept studies where we stress tested the antigen design process described in **Figure 1** to develop subunit vaccine candidates targeting the F or glycoprotein (GP) of 2 independent class I fusion protein bearing viruses: Achimota virus 2 (AchPV2) and Wenzhou mammarenavirus (WENV). AchPV2 has been isolated from straw-colored fruit bats in Ghana and serological analysis indicates possible spillover events into human populations in Ghana and Tanzania (8). WENV and closely related viruses have been found in rodents in China, Cambodia, and Thailand (9) and were recently found to be associated with an outbreak of influenza-like disease in humans (10). There are obviously no subunit vaccine candidates that have been developed against either of these viruses and there is limited published data outside of sequence information to assist development. Consequently, targeting AchPV2 and WENV was ideal for the purpose of this study and to simulate a Disease X type scenario. Importantly, the antigen design and analysis process of this study was used to optimize and aid the rapid development of a SARS-CoV-2 Sclamp subunit vaccine which is now being tested in Phase I clinical trials.

## Methods

### Molecular Cloning

To express the prefusion GP ectodomain, codon-optimized AchPV2 (Genbank: YP_009094464.1) and WENV (GenBank: MF595889.1) gene with variations including (i) substitution at the furin cleavage site, (ii) substitution at the signal peptide (SP), and (iii) truncation at C-terminal domain was generated with primers containing overlapping sequence by PCR mutagenesis using Phusion polymerase (New England Biolabs). The Achimota virus 2 F (AchVP2-F) and Wenzhao Mammarena virus GP (WENV-GP) codon-optimized amplicons ranging from AchPV2-F_1-488_ or WENV-GP_1-428_ were cloned upstream of a mammalian plasmid vector containing a HIV1281 (PDB ID 3P30) trimerisation motif according to the manufacturer’s protocol for in-fusion cloning and Stellar Competent Cells (Takara Bio). Integrated DNA Technologies (IDT) synthesized the codon-optimized geneblocks and primers. Plasmid DNA from transformed Stellar Cells and Agarose gel extractions were purified using NucleoSpin® Gel and PCR Clean-up kits and PureYield™ Palsmid Midiprep System (Promega) according to the manufacturer’s protocol and the concentration of the purified DNA was analyzed using the NanoDrop One (Thermofisher Scientific). Sequencing reactions to verify the plasmid DNA constructs were performed at the Australian Genome Research Facility and the sequencing data was analysed using the CLC Main Workbench (version 8.1).

### Recombinant Protein Expression

Soluble GP proteins were expressed using the ExpiCHO-S expression system (ThermoFisher Scientific) and in ExpiCHO-S Expression Medium (Gibco^TM^). This expression system comprises mammalian Chinese Hamster Ovary (11) cells, the most commonly used expression system for the production of biotherapeutics (12). We hypothesize that CHO-based expression of recombinant fusion proteins will result in a glycosylation profile reflecting that of the native viral protein following infection. Transfection was conducted following the manufacturer’s protocol for transient expression (ThermoFisher Scientific) using plasmid DNA at a ratio of 1 µg of DNA per 1 ml of culture volume when the culture cell density was 6 × 10^6^ cells/ml. Five to 7 days after transfection, the cells from each culture flask was pelleted following centrifugation (3,500 g at 4°C for 10 min) and the culture supernatant (SN) was sterile-filtered (0.22 µm) to perform protein purifications.

### Recombinant Protein Purification 

AchVP2-Fclamp and WENV-GPclamp proteins were purified using immunoaffinity chromatography on an ÄKTA Pure Protein Purification System (Cytiva). This was peformed using an in-house immunoaffinity chromatography column embedded with the anti-clamp monoclonal antibody (mAb) HIV1281 coupled to 1 or 5 ml HiTrap-NHS activated HP columns (Cytiva).

To purify proteins, culture SN was added to an anti-clamp affinity column pre-equilibrated with high salt PBS (PBS with 400 mM NaCl, 2.7 mM KCl, 10 mM Na_2_HPO_4_ and 1.8 mM KH_2_PO_4_, pH 7.4.) using the AKTA Pure. Bound resin was washed with 15 column volumes of high salt PBS before elution with high pH glycine buffer (100 mM glycine, 400 mM NaCl, 5 mM EDTA, pH 11.5). Eluted fractions were neutralised with a 1:1 v/v ratio of 1M Tris (pH 6.8) before concentration and buffer exchange into PBS using Merck Amicon Ultra-4 or Ultra-15 centrifugal filter units. The concentration of buffer-exchanged protein was analyzed at an absorbance of 280 nm using the NanoDrop One (Thermofisher Scientific).

### Electrophoretic Separation of Proteins 

NuPAGE 4 to 12%, Bis-Tris, 1.0 mm, mini protein precast gel (Life Technology, Australia) were loaded with 5–10 µg of purified proteins under denaturing condition using LDS Sample Buffer, following manufacturer’s protocol (BioRad). Visualization of in-gel proteins were achieved by staining with Bio-Safe™ Coomassie stain (BioRad). Gel documentation was performed using the ChemiDoc MP Imaging system (BioRad).

### Size Exclusion Chromatography (SEC) Analysis of Proteins 

To assess oligomeric state of the fusion proteins, 7.5–300 µg of purified recombinant protein was loaded onto a Superdex 200 Increase 10/300 GL size-exclusion chromatography column (Cytiva) using a 300–500 µl loop. Proteins were eluted using a mobile phase of PBS, pH 7.4 at a flow rate of 0.5 ml/min.

### Negative Stain Transmission Electron Microscopy (TEM) 

SEC puriﬁed complexes were deposited at approximately 0.01 mg/ml onto carbon-coated copper grids and stained with 1% (w/v) uranyl acetate (13) for 2 min. Grids were imaged at 120 KeV using a Hitachi HT7700. Images were collected using AXT 2kx2k CMOS. Image processing was performed using Relion3.1 (14). Contrast transfer functions of the images were corrected using CTFFIND (15). Individual particles (3,683 particles for construct Q of ACHPV2-Fclamp) in 30,000× images were selected manually followed by reference-free alignment and classification.

## Results

### Workflow to Rapid Development of Subunit Vaccine Candidates 

**Figure 2:**
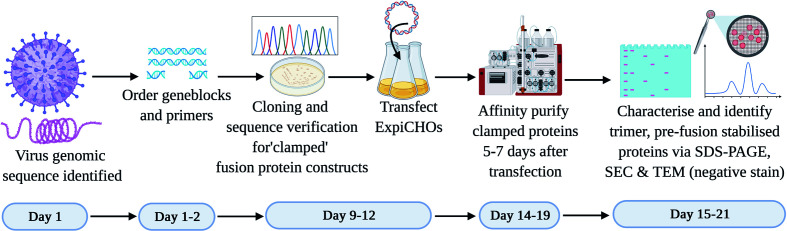
Timeline for the production of subunit vaccine candidates using the Molecular Clamp technology. Day 1–2: Following immediate availability of the virus genome sequence information, *in silico* design of ~20–30 constructs are initiated to allow for selection of optimally expressed antigens. The geneblocks and the primers arrive within 4–7 days following order placement from IDT after which cloning and transfection of the sequence confirmed constructs can be completed within 4 days. Small-scale expression of the transiently transfected cultures is completed within 5–7 days prior to affinity purification of the expressed clamped proteins using the culture supernatants. There is no need for pathogen-specific probes for the purification owing to the availability of human anti-clamp antibody embedded columns in-house. Purified proteins are analyzed *in vitro* for the expression yield, protein homogeneity on an SDS-PAGE, and the presence of the desired pre-fusion conformation by SEC and TEM (negative stain). The protein purification and *in vitro* analysis can be completed within 1–3 days.

In the context of subunit vaccine development to counteract a Disease X outbreak, it is imperative that the vaccine antigen is developed as rapidly as possible. From the many thousands of viruses reported to date, we selected two viruses that possess class I membrane fusion proteins. We established a workflow to expedite the development of a pre-fusion stabilized vaccine antigen based on the Molecular Clamp technology within 3 weeks following revelation of the virus genomic sequence (**Figure 2**). The development process involves cloning a panel of 20–30 rationally designed variants of the viral membrane fusion protein ectodomain sequence with the Molecular Clamp coding sequence inserted in place of the native transmembrane domain. Rational design of the antigen panel and cloning strategy can be completed in a matter of hours. Nucleotide geneblocks and oligonucleotide primers were ordered from Integrated DNA Technologies, Singapore, and arrived within 4–7 days for the current study.

The use of in-fusion cloning (Takara Bio) can allow us to complete the cloning within 1–2 days after receipt of the geneblock and primers. We have found that by PCR screening of colonies directly from plates using Taq DNA polymerase (New England BioLabs) provided high confidence of successful cloning and allowed us to proceed directly to protein expression without waiting for sequence conformation. *E. coli* colonies positive by PCR were grown overnight and plasmid extracted by midiprep plasmid purification (Promega). ExpiCHO-S cells (ThemoFisher) were revived from banked vials and passaged in parallel with cloning to ensure they were ready for transfection as soon as DNA was available. Each plasmid was transfected into a 25 ml culture of ExpiCHO-S cells as per the manufacturer’s instructions. Sequence verification was conducted in parallel with protein expression over 5–7 days.

Following expression, soluble protein was then purified from clarified CHO supernatant using an immunoaffinity column which was coupled with a monoclonal anti-clamp human Immunoglobulin G antibody produced in house. The purified proteins are then analysed for the expression yield by absorbance at 280 nm, homogeneity and purity by an SDS-PAGE, and for the formation of the desired trimeric, pre-fusion conformation using SEC and negative stain TEM. At the conclusion of this process the lead candidate(s) can then be selected to advance into further preclinical development including immunogenicity analysis in relevant pre-clinical models (e.g. mice) and simultaneous manufacturing process optimization. An overview of the process is shown in **Figure 2**.

### The Use of the Molecular Clamp to Develop an AchPV2 Subunit Vaccine 

**Figure 3:**
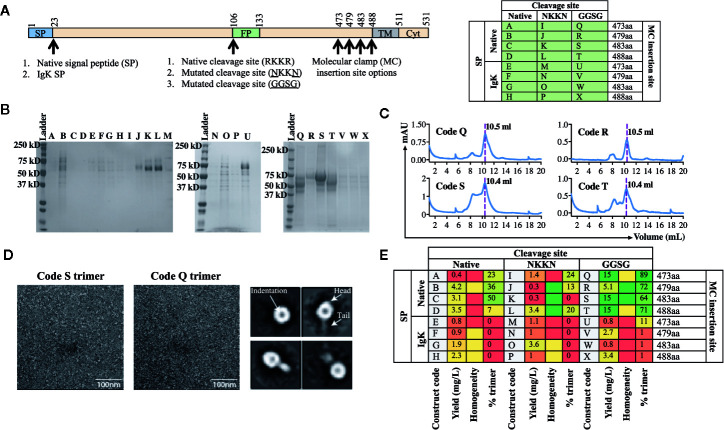
Production and analysis of AchPV2-Fclamp antigens. **(A)** The cloning strategy delineating the 24 different constructs that were desginated to be cloned for the generation of the AchPV2-Fclamp. The table shows the alphabetized (A–X) construct codes based on the different permutations of introducing signal peptides, cleavage sites, and the Molecular Clamp following removal of the transmembrane and cytosolic regions of the F. The mutations introduced to the putative native cleavage site are underlined. **(B)** SDS-PAGE analysis of the expressed AchPV2 Fclamp proteins under reducing conditions. **(C)** Representative size exclusion chromatographs for construct codes Q, R, S, and T which were the four highest expressing constructs. The dotted line overlaps with the trimer peak and the elution volume of this peak is indicated. **(D)** Representative 30,000× images of the TEM negative stain analysis of the trimeric fraction from the size excluded AchPV2-Fclamp codes S (left) and Q (middle). Representative 2D averages for Q, characteristic indentation was visible in the head domains, some particles appear with head and tail features. **(E)** Summary of the purification and *in vitro* analysis of the AchPV2 Fclamp constructs highlighting the expression yield, percentage of the purified protein in the trimeric conformation and the homogeneity of the proteins on an SDS-PAGE. For the homogeneity analysis: red shaded = least homogenous or no proteins corresponding to AchPV2-Fclamp, yellow shaded = intermediate homogeneity, and green shaded = highly homogenous.

Using the workflow strategy described above (**Figure 2**) we aimed to develop an AchPV2-F protein (class I) subunit vaccine candidate in a pre-fusion stabilized confirmation using the Molecular Clamp. Using the permutations and the parameters described in **Figure 1**, we devised a cloning strategy for a panel of 24 variant antigens that included (*i*) two SP variants (native and insertion of the IgK SP), (*ii*) three variants at the furin cleavage site (native, mutation to ^103^NKKN^106^ or ^103^GGSG^106^ to prevent Furin cleavage), and (*iii*) incorporation of the Molecular Clamp sequence at four distinct sites at the C-terminus (aa473, 479, 483, and 488). For simplicity, the resulting 24 permutations of these changes are denoted alphabetically from A–X and summarized in **Figure 3A**.

Following transient expression in ExpiCHO cell culture and purification by anti-clamp immunoaffinity chromatography, the expression yield of the purified Fclamp proteins ranged from 0.4 mg/L to as high as 15 mg/L. Protein homogeneity as determined by SDS-PAGE also differed depending on the construct with visible cleavage products detected for some constructs (**Figures 3C, E**). All constructs including the IgK SP were found to express to a much lower level than those including the native SP. However, the constructs Q, R, S, and T comprising of the native SP and the GGSG cleavage site mutation had the highest expression yield. Interestingly, mutation of the cleavage site to ^103^NKKN^106^ did not appear to prevent proteolytic cleavage and mutation to ^103^GGSG^106^ resulted in a cleavage profile that varied between constructs with distinct C-terminal lengths (**Figure 3B**). The SEC analysis showed that these protein constructs were mostly in the desired trimeric conformation: % trimer = 89% (Q), 72% (R), 64% (S), and 71% (T) (**Figures 3C, E**). We analyzed the size excluded trimer fraction (**Figure 3C**) from codes Q and S using negative TEM which suggested that the analyzed trimer fraction appeared to be in the prefusion stabilized trimeric conformation. The trimeric fraction from Q and S are highly homogeneous, both in size and shape (16 nm) (**Figure 3D**, left and middle panel****). The 2D averaging analysis showed that some particles had a globular shaped head with an indentation in the center, while some had a globular head with a short tail (**Figure 3D**, right panel****). This difference in appearance is likely caused by a different view of the same protein conformation, consistent with previous reports of the pre-fusion conformation for fusion proteins of viruses of the paramyxoviridae family (4).

### The Use of the WENV GPclamp Vaccine 

**Figure 4:**
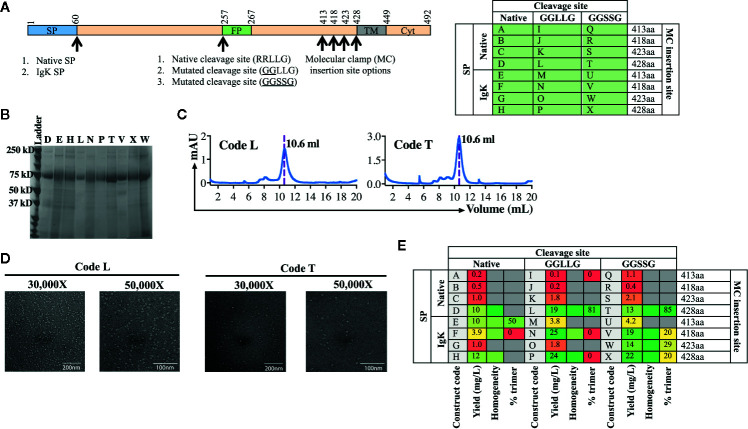
Production and analysis of WENV GPclamp antigens. **(A)** The cloning strategy delineating the 24 different WENV GPclamp constructs and the table shows the alphabetized (A–X) construct codes based on the different permutations of signal peptides, cleavage sites, and the Molecular Clamp insertion. The mutations introduced to the putative native cleavage site are underlined. **(B** **)** SDS-PAGE analysis of the 10 highest expressing WENV GPclamp proteins under reducing conditions. **(C)** Representative size exclusion chromatographs for construct codes L and T which were the two highest expressing constructs also comprising of the highest proportion of trimer among the purified GPclamp protein solution. The dotted line overlaps with the trimer peak and the elution volume of this peak is indicated. **(D)** Representative 30,000× and 50,000× images of the TEM negative stain analysis of the trimeric fraction from the size excluded WENV GPclamp codes L and T. **(E)** Summary of the purification and *in vitro* analysis of the WENV Fclamp constructs highlighting the expression yield, percentage of the purified protein in the trimeric conformation, and the homogeneity of the proteins on an SDS-PAGE. For the homogeneity analysis: red shaded = least homogenous or no proteins corresponding to WENV-GPclamp, yellow shaded = intermediate homogeneity, and green shaded = highly homogenous. Gray shaded boxes indicate when the respective protein analysis were not performed.

For the second test of the platform, we aimed to develop a subunit vaccine for WENV using the same workflow established for AchPV2 (**Figure 2**). Again we designed a panel of 24 variant antigens that included (*i*) two SP variants (native and insertion of the IgK SP), (*ii*) three variants at the cellular subtilisin kexin isozyme-1 (SKI-1)/site-1 protease (S1P) cleavage site (native, mutation to ^253^GGLLG^257^ or ^1253^GGSSG^257^ to prevent cleavage), and (*iii*) incorporation of the Molecular Clamp aminoacid sequence at four distinct sites at the C-terminus (aa413, 418, 423 and 428) (**Figure 4A**).

Following purification of the expressed proteins, the yield of the recovered GPclamp proteins ranged from 0.2 to 25 mg/L (**Figure 4E**). Insertion of the IgK SP in place of the native SP resulted in a higher yield and the longest C-terminus (aa428) also gave the highest yield. Subsequently, we prioritized the protein characterization analysis to encompass the 10 highest expressing constructs (D, E, H, L, N, P, T, V, W, and X) which expressed ≥10 mg/L of protein. While some background host cell proteins or low molecular weight degradation products were visible by SDS-PAGE, the purified antigen displayed good homogeneity and no obvious cleavage to GP1 and GP2 with either native or mutated cleavage site variants (**Figure 4B**). SEC analysis revealed a large degree of variation between constructs, with many showing the presence of significant aggregation while for constructs T and L, >80% of protein was present as soluble trimer (**Figures 4C, E**). Interestingly, while the IgK SP resulted in higher yield it appeared that the native SP was required for formation of the native soluble trimer. Following TEM analysis of the trimer fraction, constructs T and L appeared to be in the pre-fusion stabilized trimeric conformation of the protein revealing a monodisperse population with a diameter of 17 nm (**Figure 4D**). The class average showed undefined borders. This could be due to preferential binding of the samples on the EM grid or a high degree of glycosylation heterogeneity of the samples (data not shown).

## Discussion

In the midst of the COVID-19 pandemic, the importance of platform technologies for vaccine development that are applicable to rapid response has never been more evident. Notably, the majority of front-runners in the race to develop a vaccine are predominantly nucleic acid or viral vector approaches (16–18). These approaches are generally more broadly applicable, however they still need to encode and express *in situ* a target viral protein in the correct conformation that will induce an appropriate protective immune response. Subunit vaccines face their own set of challenges which are unique to each pathogen. Whereas viral vector and nucleic acid based platforms both express their target antigen within the body following vaccination, in subunit vaccines the target antigen itself must be reliably and consistently manufactured with a desired conformation that is stable throughout manufacturing, transport, and storage, right up until the moment of vaccination.

The viral surface proteins responsible for membrane fusion and viral entry are the primary target for a protective immune response, however such proteins are poorly conducive to subunit vaccine manufacture due to their inherent instability. Viral fusion proteins catalyse the merger between the viral envelope and the target cell’s membrane during viral entry. To drive this energetically unfavorable event, viral fusion proteins undergo a major conformational change from a metastable pre-fusion form present on the surface of the live virus to a more highly stable post-fusion form. Manufacture of these proteins in their inherently unstable but crucially important pre-fusion conformation is complicated by this tendency to fall toward the lower free energy post-fusion conformation. Structure-based modifications have been shown to successfully facilitate stabilization of some viral fusion proteins (4–6), and stabilization approaches previously identified for MERS-CoV Spike and SARS-CoV Spike were able to be successfully incorporated into SARS-CoV-2 Spike (19). However, while early structural data has assisted in one vaccine approach for COVID-19 (11), such structure-based antigen design is generally not conducive to a rapid response pipeline.

The proprietary Molecular Clamp platform technology we have developed has two major advantages making it uniquely suited to an emergency vaccine response. Firstly, the hyper-stable, six-helical bundle structure of the Molecular Clamp imparts sufficient stability to reliably constrain the prefusion conformation in the absence of structure-based physically incorporated constraints, and secondly, the availability of a mAb to the Molecular Clamp enaples the use of a consistent, first-pass, purification method irrespective of the viral antigen. In the current study we have outlined a process through which antigen design can be reduced down to three weeks and present two successful proof of concept studies with novel viral pathogens. The rapidness of the antigen design process also facilitates the screening of a larger panel of antigens based on the permutations described in **Figure 1** or for conducting subsequent screening if the initial study does not identify a suitable lead candidate. Furthermore, the process described for antigen design and development (**Figures 1** and **2**), was utilized to rapidly develop a SARS-CoV-2 Spike subunit vaccine, SARS-CoV-2 Sclamp (Watterson et al., under review).

Central to the process of accelerated vaccine design was the parallel cloning, expression, and characterization of a small panel of antigen designs. Our previous work, as well as the results of this study have shown that modifications at certain sites can have a large impact on the expression level, homogeneity, and the strucutural integrity of the prefusion conformation. Of equal importance are the assays used to screen candidates. These assays are well suited to a rapid assessment of key candidate biophysical and antigenic parameters and facilitates selection of a lead candidate that can proceed into further preclinical development including mouse immunogenicity analysis and simultaneous manufacturing process optimization. Of note, SEC facilitates a first-pass correlate of pre-fusion stabilization as transition to the post-fusion form results in the exposure of the hydrophobic fusion peptide and aggregate formation.

Based purely on sequence information of these relatively uncharacterised viruses and homology to more well studied relatives allowed us to select three regions to selectively target for modification (SP, cleavage site, and MPER). While we were unable to predict which of these constructs would be most successful, simple empirical analysis of the expressed products identified lead candidates. Ultimately, the native SP proved to be preferable for inclusion in both AchPV2 and WENV. Replacement of the native cleavage site with a flexible linker was also optimal for both viruses, however for AchPV2 the most truncated MPER was optimal, while for WENV the longer MPER was preferable.

Whilst the purpose of this study was to optimize the screening and rapid production of trimeric Class I fusion proteins using the Molecular Clamp in a Disease X scenario, it is important to demonstrate that the lead candidates screened are immunogenic and capable of eliciting virus neutralizing antibodies in pre-clinical models to progress into clinical trials. We are currently developing a pseudovirus neutralization assay for this purpose and to overcome this caveat of the current study. For the Molecular Clamp based subunit vaccine we developed against SARS-CoV-2, this was not an issue given the availability of live virus neutralization assays and animal challenge models shortly after the announcement of the virus sequence (Watterson et al., under review).

Stable trimer formation of expressed fusion protein ectodomains, as assessed by SEC analysis, is not definitive confirmation of the integrity of a pre-fusion protein structure. However we, and others, have empirically found that SEC confirmed trimer formation is highly indicative of a pre-fusion class I protein structure (20). Furthermore, members of the *Paramyxoviridae* have been shown in TEM to aggregate in rosettes and/or form “golf tee” shaped structures upon transition to the post-fusion conformation (21, 22), and members of the *Arenaviridae* have been shown to dissociate into monomers (23, 24). In addition, recent studies (20, 22), including our own experience with developing subunit vaccines using the Molecular Clamp for class I fusion proteins, suggest that TEM analysis confirms that the peak corresponding to the protein trimer in SEC analysis is indeed in its pre-fusion conformation.

We do not anticipate that the learnings from the optimal design of vaccine candidates targeting these viruses can be directly translated to others, unless there is a high degree of sequence homology. Therefore, the process we have defined necessitates the production of a small panel for the selection of a lead candidate. During an emergency response, any problem necessitating changes to the antigen sequence would constitute a substantial time delay, so we anticipate this emphasis on rapid screening and lead selection is likely to translante into valuable improvements during manufacture. Fortunately, we were able to undertake these two mock tests of our process during 2019. The learnings and personel training from this exercise proved invaluable to our SARS-CoV-2 response in 2020 and resulted in the generation of a Molecular Clamp stabilized subunit vaccine that is currently being tested in a Phase I clinical trial.

## Data Availability Statement

The raw data supporting the conclusions of this article will be made available by the authors, without undue reservation.

## Author Contributions

KC, DW, and PY conceptualized the project. KC edited and finalized the manuscript draft. DKW designed the experiments and the manuscript figures and wrote the initial draft. SA and JL executed the experiments, performed data analysis, and wrote the initial draft. NM led the design of TEM experiments and contributed to the drafting process of the manuscript. AI performed the TEM analysis with NM. All authors contributed to the article and approved the submitted version.

## Funding

The work was carried out with a project grant (CFP2) from the Coalition for Epidemic Preparedness Innovations (CEPI).

## Conflict of Interest

The authors declare that the research was conducted in the absence of any commercial or financial relationships that could be construed as a potential conflict of interest.
